# Documenting the Sporangium Development of the Polypodiales Fern *Pteris multifida*

**DOI:** 10.3389/fpls.2022.878693

**Published:** 2022-04-29

**Authors:** Nai-Ying Yang, Xin-Lei Jia, Chang-Xu Sui, Shi-Yi Shen, Xi-Ling Dai, Jing-Shi Xue, Zhong-Nan Yang

**Affiliations:** Shanghai Key Laboratory of Plant Molecular Sciences, College of Life Sciences, Shanghai Normal University, Shanghai, China

**Keywords:** fern, sexual reproduction, sporangium development, fluorescence imaging, spore

## Abstract

Reconstructing the development of sporangia in seed-free vascular plants provides crucial information about key processes enabling the production of spores that are important in the life cycle of these plants. By applying fluorescence imaging in intact tissues using dyes and confocal microscopy, this study aimed to reconstruct the key steps during the development of sporangia. Special emphasis was taken on the cell wall structures of tapetum and spore mother cells that have been challenged by microscopical documentation in the past. After staining the cell wall and cytoplasm using calcofluor white and basic fuchsin, the sporangium development of *Pteris multifida* was observed using confocal microscopy. The clear cell lineages from the sporangial initial cell to stalk, epidermis, inner tapetum, outer tapetum, and sporogenous cells were revealed by confocal imaging. The sporangium development improved in this work will be useful for a general understanding of fern spore formation.

## Introduction

The pteridophytes are constituted of lycophytes and ferns, both of which are spore plants (Smith et al., 2006; PPGI, 2016). Ferns are the second largest group of vascular plants (Kenrick and Crane, 1997), which provides functional comparisons with bryophytes, lycophytes, and seed plants (Harald Schneider et al., 2002; Schneider, 2013; Plackett et al., 2015). Ferns are further divided into eusporangiate and leptosporangiate (Clark et al., 2016). *Pteris multifida* Poir. belongs to the genus *Pteris* of the family Pteridaceae in the order Polypodiales of Leptosporangiatidae. Ferns include about 10,578 species, of which about 8,714 species belong to the Polypodiales. Pteridaceae has about 1,211 species, of which 250 species belong to the genus *Pteris* (PPGI, 2016). In the relatively evolved Polypodiales, the genus *Pteris* is a primitive group (Smith et al., 2006). *Pteris multifida* is native to Asia and is now widely distributed in many tropical, subtropical, and temperate regions of the world. It grows on walls, wells, and limestone crevices or under shrubs (Riefner and Smith, 2016). *Pteris multifida* is also one of the most common ornamental ferns. This species is a kind of folk herbal medicine known as Fengwei grass (Zheng et al., 2008; Ouyang et al., 2010; Yu et al., 2013).

Sporangium is the reproductive organ to produce spores. Fern plant shows great differences in sporangium morphology. Sporangium development in leptosporangiate had been carried out since Sheffield and Bell, 1979; the later decades of the 19th century (Bower, 1891; Wilson, 1958b,^1999^;

Long et al., 2016). Based on the research in some common genera, such as *Plagiogyria*, *Asplenium*, *Pteridium*, *Pyrrosia*, *Xiphopteris* of Polypodiales, fern sporangium development has been established (Bower, 1910; Warren and Wager, 1953; Wilson, 1958b,^1960^; Holttum et al., 1970; Sheffield and Bell, 1979).

Semi-thin sectioning is the major method for cellular analysis of sporangium development in fern (Wilson, 1958b,^1999^; Oldenhof and Willemse, 1999). This method may damage the tissue morphology which misleads results (Wilson, 1958b; Xue et al., 2021). During meiosis of sporangium development, the cell wall of the tapetum and the spore mother cells become extremely difficult to discern, and the actual behavior is difficult to recognize through the sections (Wilson, 1958b). With advances in microscopy technology, several fluorescence imaging methods have been developed to observe plant intact tissues. These methods provide high-resolution images *in vivo*, without sectioning and dehydrating (Truernit et al., 2008; Andriankaja et al., 2012; Sappl and Heisler, 2013; Kurihara et al., 2015; Ursache et al., 2018). Anther of seed plant is a homologous organ of the sporangium in fern. Recently, a fluorescence imaging method has been successfully used in *Arabidopsis*, showing clear cell lineages during anther development (Xue et al., 2021). Here, we established a co-staining method to observe sporangium development in leptosporangiate *Pteris multifida*. We provide clear cell lineages of the epidermis, inner tapetum, outer tapetum, and sporogenous cells of *Pteris* sporangium. These results will be helpful to the general understanding of sporangium development in ferns.

## Results

### A Co-staining Method for the Cell Lineages and Sporangium Development

**FIGURE 1:**
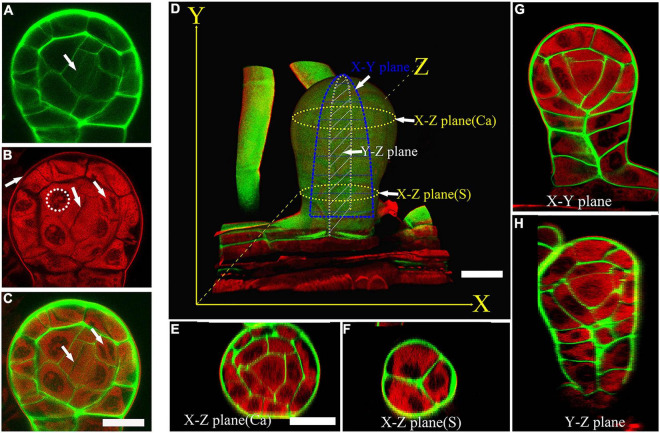
A co-staining method for the cell lineages and sporangium development. **(A)** CW staining of a sporangium. CW signals are colored green. Arrow points to the newly formed cell wall. **(A–C)** are at the same magnification. **(B)** BF staining of a sporangium. BF signals are colored red. Dotted circle points to the uncolored nuclear. The middle arrow points to the cytokinesis. The left arrow points to the outer wall of epidermal cells. The right arrow points to the nuclear division. **(C)** Merged image of **(A,B)**. The left arrow points to the newly formed cell wall. The right arrow points to the nuclear division. Scale bar = 20μm. **(D)** 3D imaging of a sporangium. CW signals are colored green. BF signals are colored red. Scale bar = 20μm. **(E)** X-Z plane of a capsule. Scale bar = 20μm. **(E–H)** are at the same magnification. **(F–H)** X-Z plane of the stalk cells, X-Y plane of the sporangium, Y-Z plane of the sporangium. CW signals are colored green. BF signals are colored red. Ca, capsule; S, stalk cell.

*Pteris multifida* is a typical species of Pteridaceae (PPGI, 2016). It has fertile leaves with sporangia in the intramarginal position (Supplementary Figure 1). To observe the sporangium development and cell lineages, we established a technique of co-staining and confocal imaging in *Pteris multifida*. Sporangia were fixed in ClearSee to improve the transparency for deep imaging (Supplementary Figure 1E; Kurihara et al., 2015). Calcofluor white (CW) stains polysaccharides of the cell wall (Ursache et al., 2018). Basic fuchsin (BF) is a commonly used red fluorescent dye for lignin, a product of the phenylpropanoid pathway (Yokoyama et al., 2007; Ursache et al., 2018). Recently, we showed that the sporopollenin wall of vascular plants also contains derivatives of the phenylpropanoid pathway (Xue et al., 2020), and BF could stain microspore sporopollenin wall. To observe both the cell lineages of sporangia and sporopollenin wall, co-staining of BF and CW was performed (Supplementary Figure 1E). The CW signals colored green clearly show the cell wall of sporangial layers (Figure 1A). The weak CW signals indicate newly formed cells in the sporangium (Figure 1A). The BF signals colored red are mainly detected in the cytoplasm of all the sporangial layers (Figure 1B), indicating that the phenylpropanoid derives might be synthesized in these cells. BF staining shows the cytokinesis of the sporangial cell, which is consistent with the CW staining showing newly formed cell walls. Therefore, the co-staining can observe the different cell layers and cell divisions in sporangium (Figure 1C). We performed a Z-stack of a series of 2-dimensional (2D) images to re-constitute the 3-dimensional (3D) structure of the sporangium (Figures 1D, and Supplementary Figure 2). The transverse light section of the sporangium is called the X-Z planes (Figures 1E,F). The periclinal light section of the sporangium is called the X-Y planes (Figure 1G). The anticlinal light section of the sporangium is called the Y-Z planes (Figure 1H). The morphology of the sporangium is observed in these light sections for a comprehensive analysis of the 3D organ structure, cell division, and developmental process (Supplementary Figure 2).

### The Sporangium Development Begins With Several Asymmetric Cell Divisions

**FIGURE 2:**
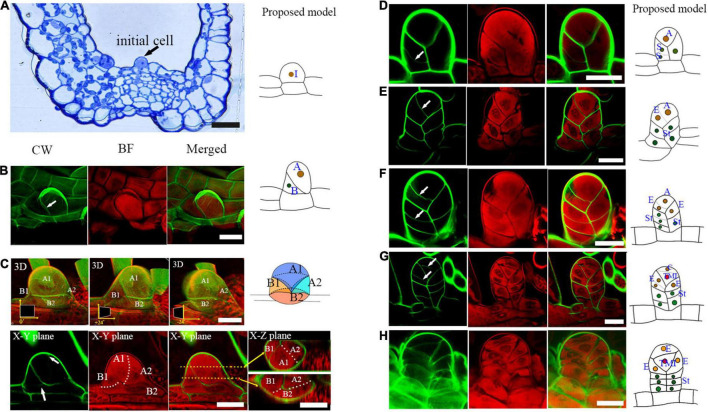
Several rounds of asymmetric cell division initiate early sporangium development. **(A)** Semi-thin section stained with toluidine blue shows a sporangial initial cell (indicated by an arrow). **(B)** A sporangial initial cell under asymmetric division. Arrow points to the cell wall **(C)**. The sporangium consists of four cells. The 3D diagram shows three rotations of 0 degrees, positive 24 degrees, and negative 24 degrees, respectively. The upper arrow points to the newly forming cell wall between the apical cell, the lower arrow points to the newly forming cell wall between the basal cell of the CW staining photo. The two white dotted lines represent the cell walls that will be formed. The two orange dotted lines represent two X-Z planes which are indicated with the two orange arrows. **(D)** Basal cells performed transverse division which formed the sporangial stalk. Arrow points to the newly formed cell wall. **(E)** The apical cell formed a new stalk cell. Arrow points to the newly formed cell wall. **(F)** The apical cell formed an epidermis cell and a new apical cell through oblique divisions. Arrows point to the newly formed cell wall. **(F)** An epidermis cell formed a stalk cell. Arrows point to the newly formed cell wall. **(H)** The tetrahedral mother initial cell formed. CW signals are colored green. BF signals are colored red. Scale bar = 20μm. **(A–H)** Proposed models are shown on the right of each inset. **(A)**, the apical cell; **(A1, A2)**, the daughter cells of the apical cell. **(A1)**, the new apical cell; **(A2)**, the third basal cell; TMI, the tetrahedral mother initial cell; **(B)**, basal cell; **(B1, B2)**, the daughter cells of basal cell; **(C)**, the cap cell; **(E)**, the epidermis cell; I, the initial cell; St, the stalk cell; 3D, three-dimensional.

To observe the sporangium development in *Pteris*, this co-staining method was used in this study. The sporangium originates from a single superficial primordial cell of the receptacle, which is called the sporangial initial cell (Figure 2A; Bower, 1891; Wilson, 1999). The outer growth of the sporangial initial cell establishes the proximal-distal axes (Y-axis in this study) of the sporangium. The formation of an oblique cell wall indicates that this sporangial initial cell undergoes an asymmetric division (Figure 2B). Thereafter, both the basal and apical cells undergo further cell expansion (Figure 2C). The second round of asymmetric cell division occurs both in the apical and basal cells. The basal cell divides into two daughter cells, occupying about 2/3 of the base of the sporangium (Figure 2C). In the apical cell, an anticlinal oblique cell division was observed (Figure 2C). This cell division produces a daughter cell and a new apical cell. The daughter cell occupies 1/3 of the base of the sporangium (Figure 2C). The medio-lateral (*Z*-axis in this study) and adaxial-abaxial axis (*X*-axis in this study) are established after these cell divisions. At this stage, the sporangium consists of four cells: a new apical cell and three basal cells (Figure 2C).

The three basal cells perform several rounds of cell division to form the three-rowed sporangial stalk (Figures 2D,E,F,H). However, only the upper layer of the sporangial stalk will undergo further cell division, and all of them are horizontal divisions (Figure 2F). The apical cell performs three oblique divisions in different directions, forming three epidermal cells and another apical cell (Figures 2E,F). Finally, this apical cell produces a cap cell and an enclosed tetrahedral mother initial cell through a transverse cell division (Figures 2G,H). The three epidermal cells and the cap cell surrounding the tetrahedral mother initial cell further develop into the sporangial epidermis (Figures 2G,H). The sporangium now consists of a stalk and a capsule (Figure 2H). The capsule is constituted of four epidermis cells and an enclosed mother initial cell (Figure 2H).

### The Tetrahedral Mother Initial Cell Generates Outer Tapetum, Inner Tapetum, and Sporogenous Cells

**FIGURE 3:**
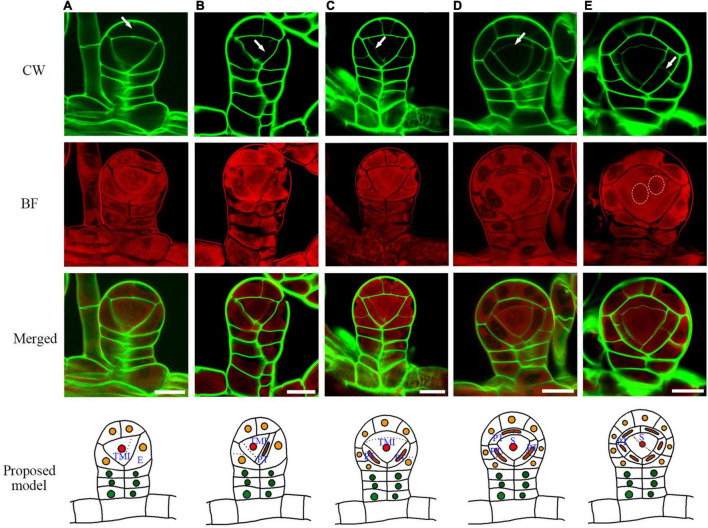
The sporangium development with the formation of epidermis, tapetum, and sporangium cells. **(A)** The cap epidermis cell of the capsule undergoes an anticlinal division. **(B)** The tetrahedral mother initial cell cuts off the first primary tapetum cell. **(C)** The tetrahedral mother initial cell cuts off the second primary tapetum cell. **(D)** Arrow points to the cell wall being formed between the primary sporogenous cell and the primary tapetum cell. **(E)** The primary tapetum cell undergoes an anticlinal division. Two dotted circles point to the nuclear division. CW signals are colored green. BF signals are colored red. Arrow points to the newly formed cell wall. Scale bar = 20μm. **(A–E)** Proposed models are shown on the right of each inset. TMI, tetrahedral mother initial cell; PT, primary tapetum cell; S, sporogenous cell; E, epidermis cell.

**FIGURE 4:**
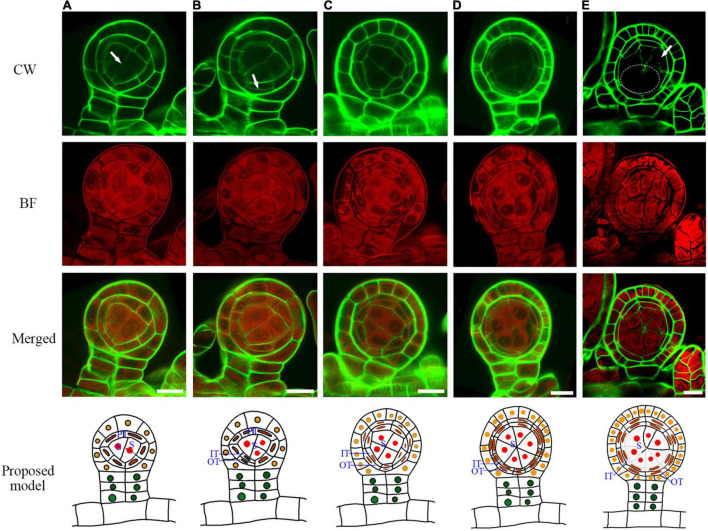
The primary tapetum was divided into two layers by the periclinal division, and the sporogenous cells were divided four times to form sixteen spore mother cells. **(A)** The sporogenous cell undergoes symmetric mitosis. Arrow points to the newly formed cell wall. **(B)** A primary tapetum cell undergoes a periclinal division. Arrow points to the newly formed cell wall.**(C)** The primary tapetal layer produces two tapetal layers through periclinal divisions.**(D)** Two tapetal layers are formed. **(E)** Some cell walls among the spore mother cells and inner tapetal cells are gradually degraded. Arrow points to the degrading cell wall between a spore mother cell and inner tapetal cell. Dotted circle points to the area where the cell walls are degraded. CW signals are colored green. BF signals are colored red. Scale bar = 20μm. **(A–E)** Proposed models are shown on the right of each inset. PT, primary tapetum cell; IT, inner tapetum cell; OT, outer tapetum cell; S, sporogenous cell.

After the establishment of the capsule, the epidermis cell layer only performs anticlinal cell division (Figure 3). The tetrahedral mother initial cell performs four rounds of oblique cell divisions to produce the primary tapetum layer and an enclosed sporogenous cell (Figures 3B–D). The primary tapetum cells undergo anticlinal cell division to increase the cell numbers before the sporogenous cell mitoses (Figure 3E). The sporogenous cell undergoes multiple symmetric mitoses to produce the spore mother cells (Figures 4A–D). Usually, each sporangium contains about 64 spores. This indicates that the sporogenous cell undergoes 4 mitoses to produce 16 spore mother cells with each of them further divided into 4 spores. The primary tapetal layer produces the outer tapetal layer and inner tapetal layer through a periclinal cell division (Figures 4A–C). The morphology of the two layers of tapetal cells is quite similar at this stage (Figure 4C). CW staining shows both sporogenous cells and tapetum cells contain cellulose walls which is similar to the regular plant cell walls during the mitotic stage (Figures 4A–C). However, the cell walls of the spore mother cells and inner tapetum cells are gradually degraded after their last mitosis (Figures 4D,E).

### The Tetrad Wall Formation and Tapetum Degradation

**FIGURE 5:**
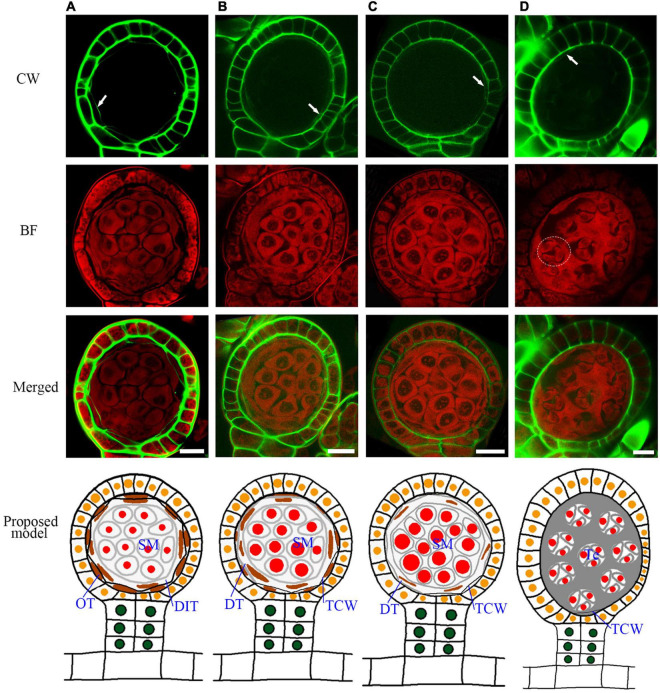
Sporangium development with tetrad formation. The spore mother cells undergo meiosis to form the tetrads, and the cell wall of the tapetum is degraded. **(A)** The spore mother cell wall has been completely degraded. The cell wall of the inner tapetum degradation. Arrow points to the wall of the outer tapetal cell. **(B,C)** The inner tapetum cells become hypertrophy. Arrow points to the wall of the outer tapetal cell. **(D)** Tetrads are formed while the inner tapetum cells are almost disintegrated. Arrow points to the wall of the outer tapetal cell. The dotted circle points to the area of one tetrad. CW signals are colored green. BF signals are colored red. Scale bar = 20μm. **(A–D)** Proposed models are shown on the right of each inset. DIT, degraded inner tapetum cell; OT, outer tapetum cell; SM, spore mother cell; Te, tetrad; TCW, tapetum cell wall.

A spore mother cell undergoes meiosis to produce a tetrad. During this process, the spore mother cell wall is gradually degraded, and some materials are accumulated outside to form a tetrad wall (Supplementary Figure 3). This cell wall transition is quite similar to *Arabidopsis* (Xue et al., 2021). After the cell wall degradation, the spore mother cells turn to round and separate from each other (Figure 5A–C). The outer tapetum becomes a very thin cell layer while the inner tapetum layer is enlarged which adheres closely to the spore mother cells (Figures 5A,B, and Supplementary Figure 4). This result indicates a functional differentiation of the inner and outer tapetum layers. After meiosis, the microspores are enclosed in the tetrad wall (Figure 5D). CW stains *Arabidopsis* tetrad wall green (Supplementary Figure 3). However, the tetrad wall of *Pteris* shows no CW signals (Figures 5A,B). This indicates that the composition of the tetrad wall of *Pteris* is different from the callose in angiosperms. The cell wall of the outer tapetum is also degraded at this stage (Figure 5D). During sporangium development, the epidermis cells undergo anticlinal division to increase the volume of the locule (Figures 5A–C). At the meiosis stage, some epidermis cells are thinner than others, suggesting that these cells are differentiated into the stomium cell (Figure 5D).

### The Lignification of the Annulus Facilitates Dehiscence After Spore Maturation

**FIGURE 6:**
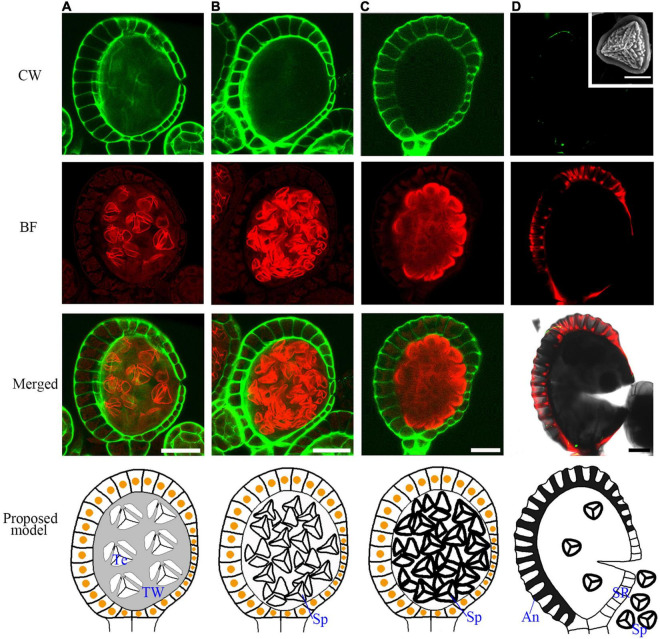
The mature spore formation and the lignification of the annulus in the epidermis for sporangium dehiscence. **(A–C)** The spores are released from tetrads in the spore sac and the spore wall is gradually thickened. **(D)** The annulus in the epidermis cells are lignified and mature spores are released from the sporangia. The SEM picture of the inset shows a mature spore. Scale bar = 20μm. CW signals are colored green. BF signals are colored red. Scale bar = 50μm. **(A–D)** Proposed models are shown on the right of each inset. An, annulus; TW, tetrads wall; SR, stomial region; Te, tetrad; Sp, spore.

At the later tetrad stage, the haploid spores are covered by a sporopollenin wall (Figures 6A,B). In seed plants, sporopollenin material is from the tapetum. However, in the *Pteris* spore, sporopollenin is gradually accumulated after the degradation of the tapetum (Figures 6A–D and Supplementary Figure 5). Thus, epidermis or spores may also provide materials for sporopollenin formation. The annulus is a special structure on the sporangium, consisting of a row of cells that are unevenly thickened on the epidermis of the sporangium. It plays a role in dehiscence and spore dispersal (Noblin et al., 2012). The BF staining shows strong signals in the thickened annulus, indicating the lignification occurs in fern sporangium (Figure 6D and Supplementary Figure 6).

## Discussion

Semi-thin sectioning is the classical tool that provides the fundamental knowledge of the developmental process of sporangia in all the land plants (Sheffield and Bell, 1979; Brown and Lemmon, 1980; Renzaglia et al., 1997; Sanders et al., 1999; van der Linde and Walbot, 2019). The two-dimensional images based on classical sectioning may miss some important information of plant cells with the wall structure. In this study, we established a fluorescence imaging method for sporangium development analysis (Figure 1). After CW and BF staining, the cell structure is visualized, which demonstrates the developmental process of both sporangium and spores even after the polysaccharide cell wall degradation (Figure 5). This easy-to-use and low-cost fluorescence imaging method avoids damage from sections and provides intact sporangium for cell lineages analysis. The anticlinal, periclinal, and transverse planes are easily analyzed in this structure, which provides novel information on sporangium development.

Through this fluorescence imaging method, the detailed sporangium developmental process of *Pteris* was visualized. The clear cell lineages from the sporangial initial cell to stalk, epidermis, inner tapetum, outer tapetum, and sporogenous cells were clearly revealed. We constructed the model of sporangium stalk formation in *Pteris*, which is different from the previous reports in other Polypodiales. The sporangial initial cell divides into an apical and a basal cell (Figure 2; Wilson, 1958b; Holttum et al., 1970). The stalk was considered to be generated from the basal cell and the capsule is generated from the apical cell in Thelypteridaceae (Holttum et al., 1970). However, in another study, basal cell and apical cell were both reported to contribute to the capsule in Grammitidaceae, Polypodiaceae, and Loxogrammaceae (Wilson, 1958b,^1960^, ^1999^). We found that the initial cell undergoes oblique division, and the formed apical cell and basal cell further undergo cell expansion (Figure 2C). Interestingly, the two daughter cells of basal cell occupy about 2/3 of the base space of the sporangium, while one daughter cell of the apical cell above is still the apical cell while the other daughter cell below occupies 1/3 of the base space of the sporangium forming a 3D structure with one cell on the top and three cells on the base (Figure 2C).

The general cell divisions in this study are consistent with the previous reports (Wilson, 1958b,^1999^; Brown and Lemmon, 1980; Rincón Barón et al., 2011; Long et al., 2016). However, the cell wall of the tapetum and the spore mother cell degraded during meiosis, which makes the actual behavior of these layers difficult to recognize in the sections of the previous investigation (Wilson, 1958b). Compared with the classical sectioning, the fluorescence imaging method can observe the process of cell wall degradation, cytoplasm transformation, and the formation of sporopollenin and lignin (Wilson, 1958a). Therefore, the sporangium development process, including cell wall degradation of the tapetum and the spore mother cells, the transformation of the outer and inner tapetum cytoplasm, formation of sporopollenin wall in spores, and the lignification of annual was visualized (Figures 4E, 5D, 6D). Based on these data, we obtained a model of the development of the sporangium in *Pteris*. In this model, cell lineages are traced, which are critical for sporangium development.

**FIGURE 7:**
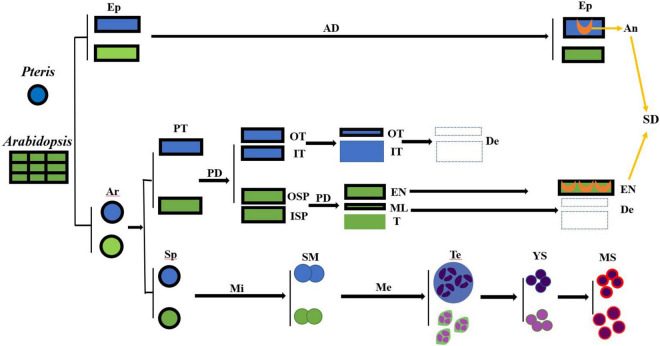
The cell lineages of the sporangia of *Pteris* and the anther of *Arabidopsis.* AD, anticlinal division; An, annulus; TMI, tetrahedral mother initial cell; De, degraded; Ep, epidermis; En, endothecium; ISP, inner secondary parietal cell; IT, inner tapetum layer; Me, meiosis; Mi, mitosis; ML, middle layer; MS, mature spores; OSP, outer secondary parietal cell; OT, outer tapetum layer; PD, periclinal division; PT, primary tapetum layer; SD, sporangia dehiscence; SM, the spore mother cells; Sp, sporogenous cell; T, tapetum; Te, tetrad; YS, young spore.

Angiosperms and pteridophytes are currently the two most prosperous vascular plants on earth. Both anthers and sporangia are used for reproductive development. The anther wall of *Arabidopsis* is divided into the epidermis, endothecium, middle layer, and tapetum. In *Pteris*, the sporangial wall contains 3 layers, including the epidermis, outer tapetum, and inner tapetum (Figure 3). The inner tapetum cell wall degrades before the meiosis of the spore mother cells, which is similar to the tapetum in angiosperms. The cell wall degradation of the outer tapetum is much later than that of the inner tapetum. Also, this cell layer becomes thinner during meiosis and tetrad stages (Figure 5), which is similar to the middle layer in angiosperm (Figure 7; Xue et al., 2021). The inner tapetum is likely sufficient to support spore development, and the middle layer of seed plants is evolved from the outer tapetum of their ancestors. The epidermis is an important cell layer to protect the sporangia in all the land plants. Most leptosporangiate ferns, including *Pteris*, have annulus in their epidermis. The lignification of the annulus is important for spore release. The epidermis of most fern plants seems to play roles of protection and spore release. In angiosperms, the epidermis plays a protection role while its lignification for pollen release is undertaken by the endothecium (Wilson, 1958b; Wilson et al., 2011; Noblin et al., 2012; van der Linde and Walbot, 2019). The sporangium development in *Pteris* and its comparison with *Arabidopsis* anther development is helpful to the general understanding of plant reproduction.

## Materials and Methods

### Plant Materials

Sporangia at different development stages were collected from *Pteris multifida* Poir. grown in the campus of Shanghai Normal University (Shanghai, China) between April 1, 2020, and October 29, 2020. Each fertile leaf has a large number of sporangia at different development stages. For each development stage, at least 3 sporangia were imaged. The voucher specimens (NO.20200401001, NO.20200701001, and NO.20201029001) are deposited in the spore function laboratory herbarium of Shanghai Normal University.

### Chemicals

Calcofluor white (CAS: 4404-43-7, Product no. F3543, Sigma) stains the primary cell wall and callose. However, the primary cell wall of the tapetum and the spore mother cells degrade during meiosis and become extremely difficult to discern (Wilson, 1958b). Basic Fuchsin (CAS: 58969-01-0, Product no. B0904, Sigma) stains the phenylpropanoid phenolics. We showed the expression of these pathway genes in tapetum (Xue et al., 2020), and this dye also stains the sporopollenin wall (Jia et al., 2021). Furthermore, these two dyes show non-overlapped emission wavelengths. Thus, we used these dyes for the staining. Toluidine Blue (CAS: 3209-30-1, Product no. 89640, Sigma).

### Tissue Preparation

ClearSee solutions were prepared by mixing xylitol powder, sodium deoxycholate, and urea in water as previously described (Kurihara et al., 2015). Sporangia were fixed in ClearSee solution, placed under vacuum for 15 min, and then were treated for two weeks, washed three times in pure water, followed by staining in a mixture of Calcofluor white (0.01M, Sigma)) and Basic Fuchsin (0.001M, Sigma) under 4 degrees in the dark for two days. The sporangia were washed with pure water, and then immediately observed by confocal scanning laser microscopy (objectives were 60x, and lasers were 405nm and 561nm). Sporangia at different developmental stages were fixed overnight in FAA [ethanol 50% (v/v), acetic acid 5.0% (v/v) and formaldehyde3.7% (v/v)], dehydrated in a graded ethanol series (50, 70, 80, 90, 95, and 100% twice), transferred to acetone (100% twice) and embedded in Spurr’s epoxy resin. Sections (1μm) were cut on a Leica UC7. Sections after staining with toluidine blue were taken photographs using an Olympus DX51 digital camera.

### Confocal Microscopy

For Calcofluor white, we used 405 nm for excitation and the emission spectra were recorded in the range of 418-468 nm. For basic fuchsin, we used 561nm excitation and emission spectra were recorded in the range of 600-650 nm. All fluorescence images were obtained with an Olympus FV3000 laser scanning microscope. The 3D reconstruction was performed by Olympus FV31S-SW.

## Data Availability Statement

The original contributions presented in the study are included in the article/Supplementary Material, further inquiries can be directed to the corresponding author/s.

## Author Contributions

Z-NY and J-SX conceived and designed the experiments. N-YY, X-LJ, C-XS, and S-YS performed the experiments. N-YY, J-SX, X-LD, and Z-NY analyzed the data. Z-NY, J-SX, and N-YY wrote the manuscript. All authors have read the manuscript and approved it for submission.

## Conflict of Interest

The authors declare that the research was conducted in the absence of any commercial or financial relationships that could be construed as a potential conflict of interest.

## Publisher’s Note

All claims expressed in this article are solely those of the authors and do not necessarily represent those of their affiliated organizations, or those of the publisher, the editors and the reviewers. Any product that may be evaluated in this article, or claim that may be made by its manufacturer, is not guaranteed or endorsed by the publisher.

## References

[B1] Andriankaja M. Dhondt S. De Bodt S. Vanhaeren H. Coppens F. De Milde L. (2012). Exit from proliferation during leaf development in *Arabidopsis Thaliana*: a not-so-gradual process. *Dev. Cell* 22 64–78. 10.1016/j.devcel.2011.11.011 22227310

[B2] Bower F. O. (1891). Is the eusporangiate or the leptosporangiate the more primitive type in the fern? *Ann. Bot.* 5 109–135. 10.1093/oxfordjournals.aob.a090629

[B3] Bower F. O. (1910). Studies in the phylogeny of the filicales. I. *Plagiogyria*. *Ann. Bot.* 20 423–450. 10.1093/oxfordjournals.aob.a089277

[B4] Brown R. Lemmon B. (1980). Ultrastructure of sporogenesis in a moss. *Ditrichum pallidum* I. meiotic prophase. *Bryologist* 83 137–152. 10.2307/3242127

[B5] Clark J. Hidalgo O. Pellicer J. Liu H. Marquardt J. Robert Y. (2016). Genome evolution of ferns: evidence for relative stasis of genome size across the fern phylogeny. *New Phytol.* 210 1072–1082. 10.1111/nph.13833 26756823

[B6] Harald Schneider K. M. P. Smith A. R. Wolf P. G. (2002). “Evolution of vascular plant body plants: a phylogenetic perspective,” in *Developmental Genetics and Plant Evolution*, eds Hawkins J. A. Bateman R. M. (Boca Raton, FL: CRC Press), 330–364. 10.1201/9781420024982.ch17

[B7] Holttum R. E. Sen U. Mrrtra D. (1970). Studies in the family Thelypteridaceae II. A comparative study of the type-species of *Thelypters schmidel*, *Cyclosorus link*, and *Ampelopteris* kunze. *Blumea* 18 195–215.

[B8] Jia X. L. Xue J. S. Zhang F. Yao C. Shen S. Y. Sui C. X. (2021). A dye combination for the staining of pollen coat and pollen wall. *Plant Reprod.* 34 91–101. 10.1007/s00497-021-00412-5 33903950

[B9] Kenrick P. Crane P. R. (1997). The origin and early evolution of plants on land. *Nature* 389 33–39. 10.1038/37918

[B10] Kurihara D. Mizuta Y. Sato Y. Higashiyama T. (2015). ClearSee: a rapid optical clearing reagent for whole-plant fluorescence imaging. *Development* 142 4168–4179. 10.1242/dev.127613 26493404 PMC4712841

[B11] Long H. Li J. Li Y. Y. Xie D. Y. Peng Q. Z. Li L. (2016). Ontogenetic characterization of sporangium and spore of *Huperzia* serrata: an anti-aging disease fern. *Bot. Stud.* 57:36. 10.1186/s40529-016-0151-9 28597446 PMC5430561

[B12] Noblin X. Rojas N. O. Westbrook J. Llorens C. Argentina M. Dumais J. (2012). The fern sporangium: a unique catapult. *Science* 335: 1322. 10.1126/science.1215985 22422975

[B13] Oldenhof H. Willemse M. I. M. (1999). Functional compartments during sporangium development in the pteridophyte *Cyrtomium Falcatum* (L.f.) Presl as expressed in tapetum function. *Plant Biol.* 1 99–107. 10.1111/j.1438-8677.1999.tb00715.x

[B14] Ouyang D. W. Ni X. Xu H. Y. Chen J. Yang P. M. Kong D. Y. (2010). Pterosins from *Pteris Multifida*. *Planta Med.* 76 1896–1900. 10.1055/s-0030-1249934 20486077

[B15] Plackett A. R. G. Di Stilio V. S. Langdale J. A. (2015). Ferns: the missing link in shoot evolution and development. *Front. Plant Sci.* 6:972. 10.3389/fpls.2015.00972 26594222 PMC4635223

[B16] PPGI (2016). A community-derived classification for extant lycophytes and ferns. *J. Syst. Evol.* 54 563–603. 10.1111/jse.12229

[B17] Renzaglia K. McFarland K. Smith D. (1997). Anatomy and ultrastructure of the sporophyte of *Takakia Ceratophylla* (Bryophyta). *Am. J. Bot.* 84:1337. 10.2307/2446132 21708543

[B18] Riefner R. E. J. Smith A. R. (2016). *Pteris Multifida* (Pteridaceae rediscovered in southern California (U.S.A.), with a key to species and notes on escaped cultivars. *J. Bot. Res. Inst. Tex.* 10 517–525.

[B19] Rincón Barón E. J. Forero Ballesteros H. G. Gélvez Landazábal L. V. Torres G. A. Rolleri C. H. (2011). Ontogenia de los estróbilos, desarrollo de los esporangios y esporogénesis de *Equisetum Giganteum* (Equisetaceae) en los Andes de Colombia. *Rev. Biol. Trop.* 59 1845–1858. 10.15517/rbt.v59i4.33190 22208097

[B20] Sanders P. M. Bui A. Q. Weterings K. McIntire K. N. Yung-Chao H. Yun L. P. (1999). Anther developmental defects in *Arabidopsis Thaliana* male-sterile mutants. *Sex. Plant Reprod.* 11 297–322. 10.1007/s004970050158

[B21] Sappl P. G. Heisler M. G. (2013). Live-imaging of plant development: latest approaches. *Curr. Opin. Plant Biol.* 16 33–40. 10.1016/j.pbi.2012.10.006 23196271

[B22] Schneider H. (2013). Evolutionary morphology of ferns (monilophytes). *Ann. Plant Rev. Online* 45 115–140. 10.1002/9781119312994.apr0489

[B23] Sheffield E. Bell P. R. (1979). Ultrastructural aspects of sporogenesis in a fern, *Pteridium aquilinum*(L.) Kuhn. *Ann. Bot.* 44 393–405. 10.1093/oxfordjournals.aob.a085748

[B24] Smith A. R. Pryer K. M. Schuettpelz E. Korall P. Schneider H. Wolf P. G. (2006). A classification for extant ferns. *Taxon* 55 705–731. 10.2307/25065646

[B25] Truernit E. Bauby H. Dubreucq B. Grandjean O. Runions J. Barthelemy J. (2008). High-resolution whole-mount imaging of three-dimensional tissue organization and gene expression enables the study of Phloem development and structure in *Arabidopsis*. *Plant Cell* 20 1494–1503. 10.1105/tpc.107.056069 18523061 PMC2483377

[B26] Ursache R. Andersen T. G. Marhavy P. Geldner N. (2018). A protocol for combining fluorescent proteins with histological stains for diverse cell wall components. *Plant J.* 93 399–412. 10.1111/tpj.13784 29171896

[B27] van der Linde K. Walbot V. (2019). Pre-meiotic anther development. *Curr. Top. Dev. Biol.* 131 239–256. 10.1016/bs.ctdb.2018.11.001 30612619

[B28] Warren H. Wager J. (1953). An asplenium prototype of the genus diellia. *Bull. Torrey Bot. Club* 80 76–94. 10.2307/2482236

[B29] Wilson K. A. (1958a). Ontogeny of the sporangia in xiphopteris serrulata and pyrrosia nuda. *J. Arnold Arbor.* 39 478–493. 10.5962/p.186020 33311142

[B30] Wilson K. A. (1958b). Ontogeny of the sporangium of *Phlebodium(Polypodium) Aureum*. *Am. J. Bot.* 45 483–491. 10.1002/j.1537-2197.1958.tb13155.x

[B31] Wilson K. A. (1960). The leptosporangium of the New Zealand fern *Anarthropteris* dictyopteris. *Contributions Gray Herbarium Harvard Univ.* 187 53–59.

[B32] Wilson K. A. (1999). Ontogeny of the sporangia of *Sphaeropteris Cooperi*. *Am. Fern J.* 89 204–214. 10.2307/1547423

[B33] Wilson Z. A. Song J. Taylor B. Yang C. (2011). The final split: the regulation of anther dehiscence. *J. Exp. Bot.* 62 1633–1649. 10.1093/jxb/err014 21325605

[B34] Xue J. S. Yao C. Xu Q. L. Sui C. X. Jia X. L. Hu W. J. (2021). Development of the middle layer in the anther of *Arabidopsis*. *Front. Plant Sci.* 12:634114. 10.3389/fpls.2021.634114 33643363 PMC7902515

[B35] Xue J. S. Zhang B. Zhan H. Lv Y. L. Jia X. L. Wang T. (2020). Phenylpropanoid derivatives are essential components of sporopollenin in vascular plants. *Mol. Plant* 13 1644–1653. 10.1016/j.molp.2020.08.005 32810599

[B36] Yokoyama A. Yamashino T. Amano Y. Tajima Y. Imamura A. Sakakibara H. (2007). Type-B ARR transcription factors. ARR10 and ARR12, are implicated in cytokinin-mediated regulation of protoxylem differentiation in roots of *Arabidopsis Thaliana*. *Plant Cell Physiol.* 48 84–96. 10.1093/pcp/pcl040 17132632

[B37] Yu C. Chen J. Huang L. (2013). A study on the antitumour effect of total flavonoids from *Pteris Multifida Poir* in H22 tumour-bearing mice. *Afr. J. Tradit. Complement. Altern. Med.* 10 459–463. 10.4314/ajtcam.v10i6.11 24311869 PMC3847384

[B38] Zheng X. D. Hu H. B. Hu H. S. (2008). Two new neolignan glycosides from *Pteris Multifida Poir*. *Indian J. Chem.* 47B 773–777.

